# Willingness to pay for oral cholera vaccines in urban Bangladesh

**DOI:** 10.1371/journal.pone.0232600

**Published:** 2020-04-30

**Authors:** Abdur Razzaque Sarker, Ziaul Islam, Marufa Sultana, Nurnabi Sheikh, Rashidul Alam Mahumud, Md. Taufiqul Islam, Robert Van Der Meer, Alec Morton, Ashraful Islam Khan, John David Clemens, Firdausi Qadri, Jahangir A. M. Khan

**Affiliations:** 1 International Centre for Diarrhoeal Disease Research, Bangladesh (icddr,b), Dhaka, Bangladesh; 2 University of Strathclyde, Glasgow, United Kingdom; 3 Bangladesh Institute of Development Studies, Dhaka, Bangladesh; 4 University of Southern Queensland, Toowoomba, Queensland, Australia; 5 Karolinska Institute, Solna, Stockholm, Sweden; 6 Liverpool School of Tropical Medicine, Liverpool, United Kingdom; University of Florida, UNITED STATES

## Abstract

**Introduction:**

Cholera is a highly infectious disease and remains a serious public health burden in Bangladesh. The objective of the study was to measure the private demand for oral cholera vaccines (OCV) in Bangladesh and to investigate the key determinants of this demand, reflected in the household’s willingness to pay (WTP) for oral cholera vaccine.

**Methods:**

A contingent valuation method was employed in an urban setting of Bangladesh during December 2015 to January 2016. All respondents (*N* = 1051) received a description of World Health Organization (WHO) prequalified OCV, Shanchol^™^. Interviews were conducted with either the head of households or their spouse or a major economic contributor of the households. Respondents were asked about how much at maximum they were willing to pay for OCV for their own and their household members’ protection. Results are presented as the average and median of the reported maximum WTP of the respondents with standard deviations and 95% confidence interval. Natural log-linear regression model was employed to examine the factors influencing participants’ WTP for OCV.

**Results:**

About 99% of the respondents expressed WTP for OCV with a maximum mean and median WTP per vaccination (2 doses) of US$ 2.23 and US$ 1.92 respectively. On the household level with an average number of 4.62 members, the estimated mean WTP was US$ 10 (median: US$ 7.69) which represents the perceived demand for OCV of a household to vaccinate against cholera.

**Conclusions:**

The demand of vaccination further indicates that there is a potential scope for recovering a certain portion of the expenditure of immunization program by introducing direct user fees for future cholera vaccination in Bangladesh. Findings from this study will be useful for the policy-makers to make decision on cost-recovery in future oral cholera vaccination programs in Bangladesh and in similar countries.

## Background

Cholera remains a serious public health burden globally and especially in regions where poverty and poor sanitation are prevalent [1]. Bangladesh has one of the largest burdens of endemic cholera, with an estimated 109,052 cases each year, and approximately 66 million people are at risk of cholera [2]. There are over 3,000–5,000 deaths annually due to cholera and high caseloads and frequent outbreaks in the country [2,3]. The endemicity of cholera in Bangladesh is demonstrated by the predictable yearly occurrence of the disease in the country’s high-risk districts and the repetitive seasonal pattern of cholera outbreaks, in spring or autumn, or both [4]. Indeed, cholera is increasingly becoming an urban disease, in Bangladesh particularly for Dhaka, the capital city of Bangladesh [5]. Dhaka has also experienced massive cholera outbreaks in the past two decades, especially during major floods in 2007 and 2009. Indeed, residents of urban slums in Dhaka are still vulnerable to cholera infection [6,7]. To address this problem, policy makers recognized that an effective vaccine and vaccination strategy are essential for urban Bangladesh [3]. The World Health Organization (WHO) recommended oral cholera vaccine (OCV) for controlling cholera outbreaks in endemic regions of the world. In 2011 cholera was declared as a global priority at sixty-fourth World Health Assembly with a specific role for introducing OCV [8].

The prevention of disease burden and death through vaccination is one of the most cost-effective and public health achievements of the 20^th^ century [9–11]. Bangladesh has experienced impressive improvements in increasing immunization coverage and a significant contribution to the reduction of childhood mortality. The Government of Bangladesh is the main driver of the immunization program where private and non-governmental organizations played strong complementary roles for vaccinating people. Urban Municipalities / City Corporation, along with local government have listed healthcare providers and established sites for vaccination, based on mainly on the population size of the wards (lowest administrative unit). However, introduction and sustainability of a new vaccine is still challenging in low resource countries as the costs of new vaccines are high relative to that of traditional vaccines and thus there is a need for prioritization [10,12]. Therefore, the financing of new vaccines represents a major hurdle for immunization programs and its success depends on global commitment, internal financing mechanisms and technical and managerial capacity of those countries [13]. Further, in order to scale up universal vaccination major financial commitments are often required from the public sector as well as from other related stakeholders [14]. Additionally, private demand for vaccine that can be purchased on the private market would give important information about financing opportunity along with public funding.

For sustainability of an immunization program including the new vaccines, the countries should consider allowing self-financing from internal household resources. In the other words, charging a private domestic contribution for this new vaccine would be an option [13]. The WTP method has been proven to be a standard tool for valuation of the private demand for future vaccines [15]. Our overall objective of the study was to capture the WTP for new generation OCV if available in private market of Bangladesh, considering the household perspectives. We additionally intended to find the determinants of demand for cholera vaccine. As there is no current available cholera vaccines in the Expanded Program on Immunization (EPI), estimation of demand for vaccination and its determinants are expected to be useful for the government and policy makers to adopt long term financing strategies and design future vaccination programs in a sustainable way by adding additional resources with a given public budget. Therefore, this findings might be useful for the policy-makers to make decisions on cost-recovery in future oral cholera vaccination programs in Bangladesh.

## Materials and methods

### Methodology

To elicit respondent’s WTP Contingent Valuation Method (CVM) was used [16]. The CVM is a standard and accepted technique of stated preferences for capturing maximum WTP and was originally developed in the area of valuing environmental benefits [17]. However, in the vaccination area where the population is familiar with the potential benefit of vaccination such as avoiding cases, economic costs, pains and suffering, CVM is particularly suitable [18]. A systematic review study indicated that CVM act as a promising tool for capturing the demand for childhood immunization in many low-and middle income countries [19]. In the healthcare sector, CVM is recommended if respondents know what they are paying for [18,20]. In this study, we used open-ended bidding game techniques as it produces unbiased estimates since no particular response is promoted [21], and produces the least conservative estimates compared to other available techniques [22]. In open-ended valuation, individuals are asked to state their willingness to pay, as in a bidding game [23], and then depending on the answer, the bid is lowered or raised until reaching the respondent’s maximum willingness to pay. However, starting-point bias and anchor bias are often associated with the bidding game techniques [24,25]. In order to minimize such bias, the starting bid was taken from a pretest of the household survey and in consultation with local residents. We conducted proper training on data collection process and used open ended questions to mitigate such biases [26]. It should be noted that some previous studies used open-ended bidding game technique arguing that such bias was not observed on WTP outcomes [27,28].

### Study settings and sample

The study was conducted under the umbrella study of Gavi funded Vaccine Investment Strategy (VIS) learning agenda for oral cholera vaccine with the killed whole cell oral cholera vaccine, Shanchol^™^ (manufactured by Shantha Biotechnics, in Hyderabad, India). This study primarily aimed to assess the preventive impact, demand, acceptability, uptake, feasibility, and cost-effectiveness of a two-dose regimen of OCV targeting children from 1 to 14 years in high risk urban areas (Kamrangirchar, Hazaribagh and Rayer Bazar) of Bangladesh. Phase II clinical trials of the whole cell bivalent vaccine Shanchol^™^ in Vietnam and India and in Bangladesh have shown that this vaccine is safe and immunogenic in both adults and children [29–31]. The latest WHO Fact sheet indicated that Shanchol^™^ gives approximately 65% protection against cholera for up to 5 years following vaccination in endemic areas [32]. A cross sectional household survey was conducted from December 23, 2015 to January 16, 2016 before the cholera vaccination trial. The sample size was drawn based on earlier study in the same country context, it was found that 74% of respondents decided to purchase oral cholera vaccine for their family members [33]. In this context, the following equation was applied for the sample size calculation for WTP study:

$$n=\frac{Z^{2}P(1-P)}{d^{2}}=\frac{{\left(1.96\right)}^{2}(0.74)(1-0.74)}{{\left(0.03\right)}^{2}}=821$$

Where, n = sample size to be calculated, p = proportion having the characteristic being measured (0.74), Z = value of normal distribution at 95% confidence level (1.96), d = tolerable standard error (0.03). We assumed at 20% non-response rate during household survey therefore at least 986 households were required for this survey. The households were randomly selected from the study area, and the respondents were the household head or the major economic contributor of the household if household head was not available.

### The survey instruments

A paper-based survey instrument (questionnaire) was developed and implemented by the data collectors under the supervision of the research team. The data collectors were pre-trained in CVM survey according to the guidelines recommended in Whittington’s review of CV practices in developing countries [34] and the questionnaires were translated into the local language (Bangla) in order to maintain consistency. The survey tool is validated earlier in the context of Bangladesh [33]. The pre-test survey of the instruments was conducted in the community before the original survey to refine the language and determine respondents’ views of possible vaccine prices to offer.

The survey instrument was approved by the Research Review Committee and the Ethical Review Committee of the Institutional Review Board of the International Centre for Diarrhoeal Disease Research, Bangladesh (icddr,b). The instrument has seven sections relevant to the analysis (see S1 File). The first section recorded the respondent’s background information followed by the respondent’s informed written consent and the relationship with the particular household. Section 2 gathered the demographic information of household members along with economic status such as income, expenditure of the households. Section 3 contained the questions regarding respondent’s perceptions and knowledge about cholera. This section also discussed how cholera was contracted and their previous experience with cholera. The next section recorded understanding about vaccine and vaccination in general and about cholera vaccines in particular. Section 5 introduced the contingent valuation scenario of cholera vaccine, including the descriptions of the available Shanchol vaccines, its effectiveness and the duration of protection. Next some questions were administered in order to test respondent’s understanding about the effectiveness of proposed vaccine [35,36]. Section 6 contained the valuation questions that were used to estimate WTP for OCV for household’s member and for individual protection against cholera infection. The seventh section recorded interviewer’s observations on visible conditions of the home and opinions on the quality of the interview.

### Data collection and analysis

Data were collected through face-to-face interviews at their households by trained and experienced data collectors. The respondents were either the head of households or the economic contributor to the family. Data were entered into Microsoft Excel 2007, and all entries were manually double-checked and verified by the investigators. Before analysis, missing answers and outliers were systematically verified. Descriptive statistics were employed to analyze and summarize the data using various variables. Results are presented as a mean and median WTP with standard deviations and at 95% confidence interval, in Bangladeshi currency (BDT = Bangladeshi Taka) applying the exchange rate (US$ 1 = BDT 78) during the data collection year. Age-specific (under-five, 5 to 14, 15 to 45, 46 to 64 and 65 and above) and household-specific hypothetical demand was constructed using the proportion of respondent stating WTP and the amount of WTP for OCV [37,38]. The proportion of population who expressed the WTP of particular vaccine at the amount indicated used as a proxy of the quantity of vaccines ‘purchased’ at the indicated price [39]. Two econometric models were used in the analyses; model I was the respondent’s WTP for oral cholera vaccine and model II for the households WTP of all household members (including respondent). Natural log-linear regression model was used to examine factors influencing participants’ WTP. The data normality assumption was tested graphically and the Cook-Weisberg heteroscedasticity test was performed [40]. Power transformation was used to achieve the validity of the assumptions as the data violate both normality and heteroscedasticity assumptions. In order to obtain a suitable power transformation of the predicted variables Tukey’s ladder of power was used, from which the natural log transformation seems to be approximated to achieve the validity of the assumptions of normality [41]. The Variance Inflation Factor (VIF) was also used to check the multicollinearity among the predictors. All data analyses were performed using statistical software Stata/SE 13 (StataCorp, College Station, TX, USA).

## Results

### Participants’ characteristics

Background statistics are summarized in Table 1. A total of 1,051 households were surveyed from the study area at 100% response rate. The average age of the sampled population is about 33 years, most of the respondents were female (80%) and married (93%) Most of the respondents (69%) completed the primary and secondary education and approximately 24% had no formal schooling, respectively. Average household size was 4.62 persons and approximately 12% and 25% of the households had under-five children and young children aged 5 to 14 years old, respectively. The average monthly household income was BDT 16,780 (US$ 215.13). The average healthcare expenditure (last three months) was BDT 4,883 (US$ 62.60). More than half of the households (59%) shared a rented house while only 17% of respondents had their own house. Most of the respondents (84%) indicated that the floor of their household was made of cement and bricks (11%). Only few of them (2%) reportedly lived in soil/mud-based floor.

**Table 1:** Background characteristics of respondents, Dhaka, Bangladesh, 2015–16.

Subject	Variables	n (%)	(95% CI)
**Respondent characteristics**	**Sex of the respondent**		
	*Male*	203 (19.31)	(17.04, 21.82)
	*Female*	848 (80.69)	(78.18, 82.96)
	**Age (years)**		
	*≤ 29*	449 (42.72)	(39.76, 45.74)
	*30–39*	339 (32.25)	(29.49, 35.15)
	*40–49*	173 (16.46)	(14.34, 18.83)
	*50 and above*	90 (8.56)	(7.01, 10.42)
	**Marital status**		
	*Married*	980 (93.24)	(91.56, 94.61)
	*Others (unmarried*, *widow*, *divorce*, *separated)*	71 (6.76)	(5.39, 8.44)
	**Educational status**		
	*No formal education*	251 (23.88)	(21.40, 26.56)
	*Primary education*	374 (35.59)	(32.74, 38.53)
	*Secondary education*	352 (33.49)	(30.70, 36.41)
	*Higher secondary & above*	74 (7.04)	(5.64, 8.76)
**Household characteristics**	**Household size**		
	*Less than 4*	255 (24.26)	(21.76, 26.95)
	*4 to 5*	571 (54.33)	(51.30, 57.33)
	*More than 5*	225 (21.41)	(19.03, 24.00)
	**Floor materials**		
	*Mud/Soil*	14 (1.33)	(0.79, 2.24)
	*Cement*	883 (84.02)	(81.67, 86.11)
	*Tiles*	18 (1.71)	(1.08, 2.70)
	*Brick*	117 (11.13)	(9.37, 13.18)
	*Others*	19 (1.81)	(1.16, 2.82)
	**Types of home**		
	*Own house*	177 (16.84)	(14.69, 19.23)
	*Rented house in slum*	126 (11.99)	(10.16, 14.10)
	*Government Residence*	12 (1.14)	(0.65, 2.00)
	*Individual separated house*, *well condition*	49 (4.66)	(3.54, 6.12)
	*Rented flat/house (shared with others)*	622 (59.18)	(56.18, 62.12)
	*Individual separated house*, *not well condition*	40 (3.81)	(2.80, 5.15)
	*Others*	25 (2.38)	(1.61, 3.50)
	**Any healthcare expenditure in last 3 months**		
	*Yes*	932 (88.68)	(86.61, 90.46)
	*No*	119 (11.32)	(9.54, 13.39)
	**Income quintile**		**Mean ± SD (95% CI)**
	*Poorest quintile (*≤*9*,*000)*	245 (23.31)	6,741 ± 1,879 (6,504, 6,977)
	*2nd quintile (9*,*001–12*,*000)*	209 (19.89)	10,843 ± 949 (10,713, 10,972)
	*3rd quintile (12*,*001–15*,*000)*	206 (19.60)	14,404 ± 830 (14,290, 14,518)
	*4th quintile (15*,*001–20*,*000)*	183 (17.41)	18,458 ± 1,577 (18,228, 18,688)
	*Upper quintile (> 20*,*000)*	208 (19.79)	35,450 ± 24,916 (32,044, 38,856)

### Perception and attitude towards cholera and vaccines

Approximately 76% of the respondents mentioned that they heard about cholera infection and about 80% of the respondents believed that cholera was very serious especially for the under five children compared to other age groups (Table 2). However, half of the respondents (52%) were not sure about the risk of cholera in their community. About 29% of the respondents reported that at least one of the household members had suffered from cholera previously and 1% reported a household member died due to cholera infection. Another 26% of the respondents knew someone other than a household member who had suffered from cholera and 12% of the total respondents knew someone outside their household who died due to cholera disease. Almost all of the respondents (90%) had taken at least one vaccine in the past.

**Table 2:** Perception and attitude towards cholera and cholera vaccine (n = 1,051).

Variables	n (%)	95% CI
**Heard about cholera**		
*Yes*	797 (75.83)	(73.15, 78.33)
*No*	254 (24.17)	(21.67, 26.85)
**Perceived risk of cholera in community**		
*Not much likely*	292 (27.78)	(25.15, 30.58)
*Likely*	189 (17.98)	(15.77, 20.43)
*Most likely*	21 (2.00)	(1.31, 3.05)
*Don't know/Not sure*	549 (52.24)	(49.21, 55.25)
**Perceived risk of cholera among age groups**		
*0–5 year*	405 (80.68)	(76.97, 83.91)
*6–10 year*	33 (6.57)	(4.71, 9.11)
*11–14 year*	8 (1.59)	(0.80, 3.16)
*15–19 year*	8 (1.59)	(0.80, 3.16)
*20–64 & 65+ year*	48 (9.56)	(7.27, 12.47)
**Perceived severity of cholera among age group (0–5) year (multiple response)**		
*Very severe*	96 (23.70)	(19.75, 28.04)
*Severe*	91 (22.47)	(18.83, 27.01)
*Not very severe*	193 (47.65)	(42.70, 52.42)
*Don't know/Not sure*	25 (6.17)	(4.19, 8.97)
**Someone in household has had cholera**		
*Yes*	307 (29.21)	(26.53, 32.04)
*No*	744 (70.79)	(67.96, 73.47)
**Someone in household had died having cholera**		
*Yes*	11 (1.05)	(0.58, 1.88)
*No*	1040 (98.95)	(98.12, 99.42)
**Know someone who has had cholera (outside households)**		
*Yes*	278 (26.45)	(23.87, 29.21)
*No*	773 (73.55)	(70.79, 76.13)
**Know someone who has died having cholera (outside households)**		
*Yes*	128 (12.18)	(10.33, 14.30)
*No*	923 (87.82)	(85.70, 89.67)

Considering the effectiveness of the cholera vaccine, approximately 89% of the respondents believed that cholera could be prevented by the cholera vaccine while 14% believed that the cholera vaccine could protect them from risk of death. Of all respondents, 4% believed that cholera vaccine might reduce their treatment cost and avert sick days (3%) due to cholera infection. However, 6% were still not sure about the effectiveness of cholera vaccine. After being given the information and explanation of the OCV effectiveness we tested the understanding of the respondents in a structured way [36]. All of the respondents understood the descriptions of the vaccine effectiveness. Approximately 92% of the respondents gave the answer correctly to the four questions designed to test the understanding of vaccine effectiveness supplementary material. The data collectors explained the vaccine effectiveness description again and retested to 8% of the respondents who did not answer correctly. Finally, in total 97% respondents understood the effectiveness concepts after this second attempt.

### Willingness to pay for cholera vaccine

WTP values are shown in Table 3. The mean and median WTP for OCV per vaccination (2 doses) was estimated to BDT 174 (US$ 2.23) and BDT 150 (US$ 1.92) respectively for protection of the respondent against cholera infection. On the household level with an average number of 4.62 members, the estimated WTP was US $10 (mean) and US$ 7.69 (median) which represents the perceived private economic benefits to a household of vaccination against cholera. Among the total respondents (N = 1,051), approximately 99.4% were WTP for the vaccines for their own protection, and 99.8% reported they would purchase the vaccine for their household members. Financial unaffordability was the main reason for those who did not agree to pay for oral cholera vaccine. The estimated mean WTP per person for under-five children was slightly higher than other age groups (Table 3). Males had a higher WTP than females (BDT 176.98 or US$ 2.27 vs BDT 170.87 or US$ 2.19). A general socioeconomic gradient was observed in WTP, meaning that the richer socioeconomic groups were WTP more, with a slight exception in the 4^th^ quintile.

**Table 3:** Household’s willingness to pay for future cholera vaccine Dhaka, Bangladesh, 2015–16.

Variables	n	Mean WTP ± SD	Median	Interquartile range IQR	5th percentile	95th percentile	P-value
**Age groups**							
Up to 4	579	182.76 ± 133.89	150	100	50	400	0.125
5 to 14	1,200	168.86 ± 129.53	150	100	50	400	
15 to 45	2,455	176.13 ± 135.91	150	100	50	400	
46 to 64	370	164.62 ± 115.96	125	100	50	400	
65 and above	109	166.61 ± 111.94	150	100	50	400	
**Sex**							
Male	2,392	176.98 ± 139.33	150	120	50	400	0.056
Female	2,321	170.87 ± 124.20	150	100	50	400	
**Income quintile**							
Poorest quintile (≤ 9,000)	949	152.19 ± 92.27	130	100	40	300	0.000
2nd quintile (9.001–12,000)	1,016	160.43 ± 158.02	128	100	50	300	
3rd quintile (12,001–15,000)	867	173.45 ± 111.95	150	120	50	350	
4th quintile (15,001–20,000)	965	171.60 ± 121.85	150	100	50	500	
Upper quintile (20,000+)	916	214.55 ± 153.36	200	200	50	500	
**Household size**							
Less than 4	759	199.44 ± 181.12	150	150	50	400	0.000
4 to 5	2,455	169.86 ± 121.45	150	100	50	400	
More than 5	1,499	167.81 ± 117.27	125	100	50	400	
**Household WTP, BDT**	1,049	781.62 ± 631.99	600	550	190	2,000	
**Per capita WTP per OCV, BDT**	4,713	173.97 ± 132.12	150	100	50	400	

*BDT = Bangladeshi Taka * OCV = Oral Cholera vaccine

### Household demand for OCV

**Fig 1:**
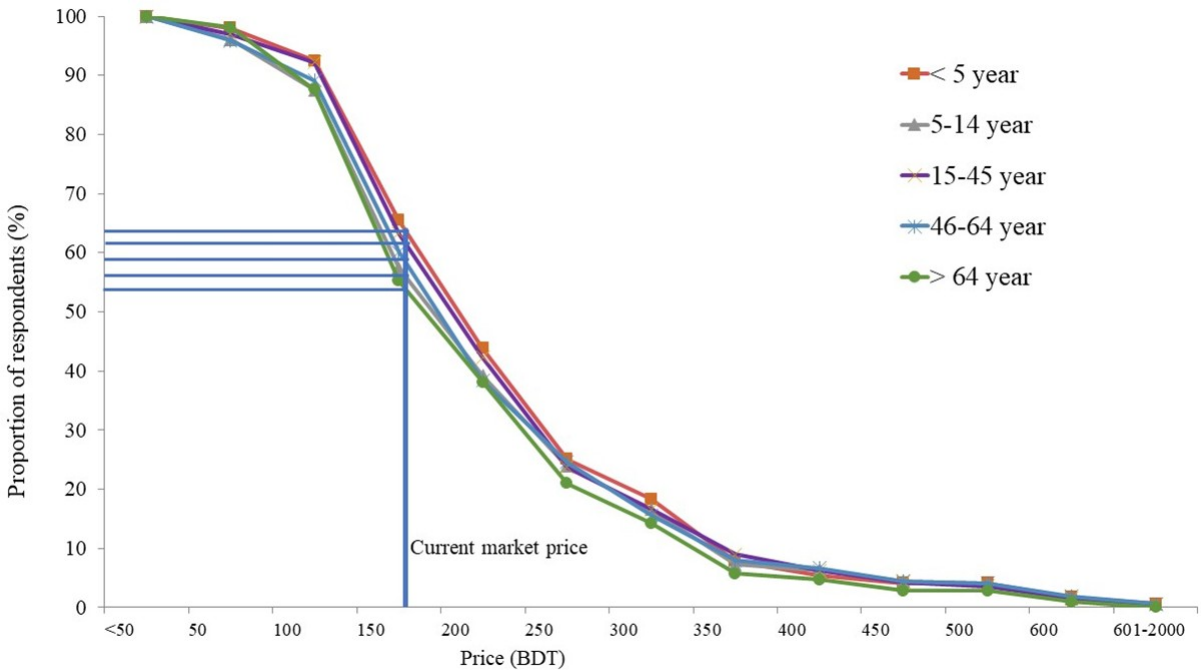
Age-specific demand for OCV, Dhaka, Bangladesh, 2015–16.

**Fig 2:**
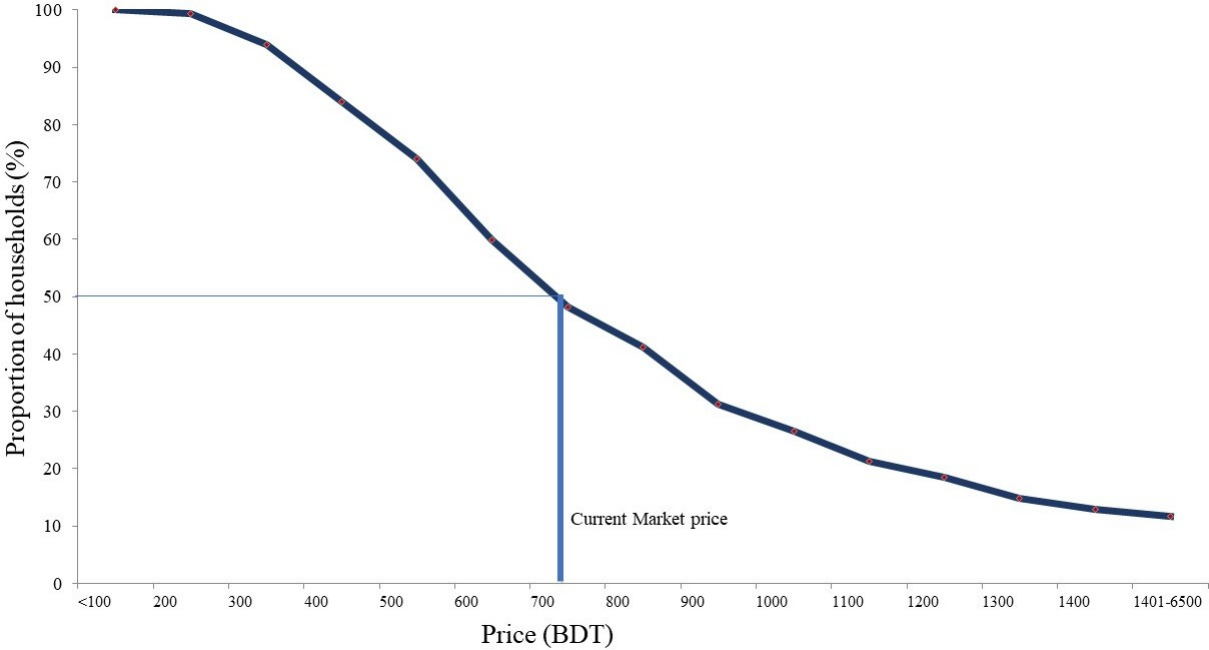
Household demand for OCV, Dhaka, Bangladesh, 2015–16.

The age-specific demand and household demand for OCV have been illustrated in Fig 1 and Fig 2 respectively. We found that the hypothetical demand for cholera vaccine is slightly higher for under-five children than people at age 15 years and above. The demand curve shows the share of households at different levels of WTP for people at different ages (Fig 1).

Fig 2 shows a negative relationship between WTP and the proportion of households WTP those specific amounts meaning that higher proportion of households are willing to pay at lower level of vaccine price. At the current market price of US$ 9.24 for vaccinating an entire household, 50% households would prefer to vaccinate themselves (Fig 2).

### Factors associated with the willingness to pay

In Table 4, the natural log-linear regression model revealed that a number of factors were significantly associated with the respondent’s WTP for protecting him-/herself and all household members from cholera infection. The factors are sex of the respondents, his/her occupation, knowledge about cholera and oral cholera vaccine, household income, size of the households and age composition of household members. Considering the sex of the respondents, males had significantly higher WTP than females and were willing to pay 15% more for himself and approximately 18% more for their households (Table 4). The employed respondents reported lower amount of WTP than unemployed. Those who were exposed for cholera in past, intended to pay more for themselves and the household (9.4% and 10.5% respectively) than those who did not have such experience. We observed that WTP was higher in the households where the number of under-five children, children aged 5 to 14 years and number of adult household members in the household increases. One under-five child will lead to 15% more WTP for household and 7.2% and 8.3% more for children aged 5–14 years and adults (>14 years) respectively. The size of the households is one of the significant negative factors on respondent WTP and larger households were negatively associated with WTP. Household with 4 to 5 members had 11.6% less WTP for respondent compared with smaller household (<4 members).

**Table 4:** Factors influencing on willingness-to-pay (WTP as a natural log form) for oral cholera vaccine, Dhaka, Bangladesh, 2015–16.

Parameters	Descriptions	Co-efficient (Standard Error)	
		Respondents’ WTP	Households’ WTP (including respondent)
**Age of respondent (years)**			
30–39	Ref: Less than 30 years	-0.01 (0.05)	0.03 (0.05)
40–49		-0.04 (0.07)	0.00 (0.07)
50 and above		-0.11 (0.09)	-0.08 (0.09)
**Sex of respondent**			
Male	Ref: Female	0.15** (0.07)	0.18** (0.07)
**Respondent educational status**			
No formal education	Ref: Higher secondary & above	-0.04 (0.09)	-0.05 (0.09)
Primary education		0.01 (0.08)	0.05 (0.09)
Secondary education		0.05 (0.08)	0.09 (0.08)
**Respondent Occupation**			
Employed	Ref: Unemployed	-0.12** (0.06)	-0.12** (0.06)
**Other variables**			
Received any vaccine	Ref: Yes	0.01 (0.07)	-0.02 (0.07)
Someone in household has had cholera	Ref: Yes	0.09** (0.04)	0.10** (0.05)
Someone in household had died having cholera	Ref: Yes	0.29 (0.20)	0.26 (0.20)
Know someone has had cholera (outside HH)	Ref: Yes	0.14*** (0.05)	0.16*** (0.05)
Heard about cholera	Ref: Yes	0.05 (0.05)	0.03 (0.05)
Cholera is common in community	Ref: Yes	0.01 (0.07)	-0.02 (0.07)
Child (<5 years) is more vulnerable for cholera	Ref: Yes	-0.04 (0.07)	-0.05 (0.07)
Healthcare Utilization	Ref: Yes	0.04 (0.06)	0.05 (0.06)
Number of U5 children	Continuous	0.03 (0.04)	0.14*** (0.04)
Number of child age 5 to 14	Continuous	-0.03 (0.03)	0.07** (0.03)
Number of adult members (>14 years)	Continuous	-0.04 (0.03)	0.08*** (0.03)
**Household size**			
4 to 5	Ref: Less than 4 members	-0.11 (0.06)	0.08 (0.06)
More than 5		-0.14 (0.11)	0.15 (0.11)
**Income quintile (BDT)**			
2^nd^ quintile	Ref: 1^st^/Poorest quintile	0.05 (0.06)	0.05 (0.06)
3^rd^ quintile		0.17**(0.06)	0.20*** (0.06)
4^th^ quintile		0.15** (0.07)	0.17** (0.07)
Upper quintile		0.36*** (0.07)	0.38*** (0.07)
Intercept	Constant	5.30*** (0.15)	6.07*** (0.15)
N		1,045	1,049
Adjusted R-square		0.054	0.164
Mean VIF		2.23	2.23
F-value, (Prob > F)		3.38***	9.20***

***significant at 1% risk level

**significant at 5% risk level

#Percentage change of WTP explained by (eβ-1)*100

Our model showed that household income was significantly positively associated with the both respondent’s and household’s WTP. Respondents from higher income households are willing to pay more compared to respondents from lower income households for their own protection as well as for their household protection. For own protection, respondents from the 3^rd^, 4^th^ and 5^th^ quintiles were willing to pay 18.5% (p <0.05), 16.1% (p<0.05), and 43.3% (p<0.001) more compared to respondents from lower income households (poorest quintile). Considering the household protection against future cholera cases, it was observed that the 3^rd^, 4^th^, and 5^th^ quintile’s WTP were 22.1% (p<0.001), 18.5% (p<0.05), and 46.2% (p<0.001) more than the poorest quintile.

## Discussion

The study was conducted in order to assess the average maximum WTP for a future cholera vaccine and its associated determinants among the household heads and their household members in an urban area of Bangladesh. Our study found that the per capita maximum WTP was BDT 174 (US$ 2.23) for OCV use against cholera disease. On the household level, each household was ready to invest BDT 782 (US$ 10.02) for purchasing the cholera vaccine to protect their members from cholera cases.

The study demonstrated that most of the respondents (98%) reported their willingness to purchase OCV for the protection of their own and their household members if financially affordable. WTP was found to be higher (98%) in urban areas in comparison with rural areas of Bangladesh as found in another study, where 75% of the responded of the rural residents were interested in OCV [33]. Such difference between urban and rural areas could be explained by the disparity of financial affordability between urban and rural people where the former were better-off. However, since Bangladesh has recently been upgraded as a lower- middle income country and poverty has declined substantially, we may expect more people, also in rural areas, to be interested to purchase OCV than in the past [42]. Our estimation supported that the households with members of age under five years were willing to pay more than any other age groups. Such findings were also observed in the rural context in Bangladesh [33]. Since it was observed that the young children were more vulnerable to cholera in Bangladesh, higher demand of OCV was thus expected [3]. Considering the current market price of OCV (US$ 2.00), the vaccination coverage is higher for under-five children (68%) than age 15 years and above. From the experience of an earlier study conducted in this setting, it was observed that the household invested more money on cholera infected children than adult members for seeking treatment [6].

Our log-linear regression model suggested that the male respondents had significantly higher WTP than the females, which was in line with findings of other studies as male often predominantly deal with the financial matters including healthcare expenditure of the household [33,43,44]. However, such relationship was not always observed [45].It was further revealed that the respondents with past experience in cholera and cholera-related deaths expressed higher WTP both for self-protection and protection of the household members. This finding was supported by earlier studies which indicated that the household risk aversion is a crucial influencing factor for demand for future OCV [33,46]. However, on the contrary, there were evidence that prior awareness of disease or having a personal history of a disease did not always lead to higher WTP [47–50]. Unlike other study, we observed that employed respondents reported lower WTP than unemployed person as income earner appeared to be much concerned about other household expenditure [51]. Therefore, further research on this topic should be required in order to provide a better explanation. Household income significantly positively affected both respondent’s and household’s WTP which was consistent with the theoretical concept of positive income elasticity that wealthier families purchase more cholera vaccines than low-income households [33,46,52]. This was crucial for policy implication in Bangladesh where 67% of total healthcare expenditures were borne by out-of-pocket payments in absence of social health insurance and the poor people were often unable to afford adequate healthcare [53].

A free-of-cost supply of OCV to a typical member of the population in this study would bring a perceived economic benefit of 781.6 BDT corresponding to the average WTP of the households. In an earlier study, we found that full vaccination of an individual cost BDT 165.36 (US$ 2.12) which included freight charge, transportation and imported price of vials [54]. The individual WTP for OCV (BDT 174 or US$ 2.23), observed in this current study, exceeded the costs of vaccination, which indicated the economic viability of OCV in a market condition. It could be argued that the delivery costs of OCV would increase the vaccination costs [54]. Such additional costs could be covered by an incremental cost to the EPI program of Bangladesh, funded preferably by pooled fund (e.g., taxes).

Recently a well-known pharmaceutical company indicated that the production of the cholera vaccine might be possible at a cost below BDT 78 (US$ 1) in Bangladesh [55]. Considering such a statement, we calculated the total costs of vaccinating a household to be BDT 720 (US$ 9.23) at which price approximately 50% of the households of Bangladesh would be able to purchase OCV from private market. For the full coverage of OCV in the country, pooled fund (e.g., taxes) could be additionally used for subsidizing the households with lower maximum WTP. It was also observed that, even at higher vaccine prices, there was demand for OCV among the wealthier households. Indeed, the poor people would be also benefit from vaccination of wealthier populations because of herd immunity [33,56]. Based on the economic condition of the country and demand for OCV as per our current study, we recommend that a sustainable financing method could be developed where pooled fund such as tax and revenue from sold vaccines in private market would be used jointly.

There are some limitations of the current study that need to be considered in interpreting the results. In applying contingent valuation techniques, a possible source of bias might arise from the fact that respondents are not purchasing the vaccine in the practical context but rather hypothetically [57]. Again due to resource constraints we did not introduce time-to-think approach [33]. Further, we did not introduce herd immunity in contingent valuation scenario, so our results underestimate the true value of this particular vaccine. We did not validate the demand for OCV using the travel cost approach where earlier studies indicated that the private demand for OCV was low because of household cost such as transportation and time cost was incurred due to receiving the vaccine [58]. All of the above represent possible avenues for further research on this topic.

## Conclusions

Our research provided evidence on the perceived demand for OCV, suggesting that the households may not wait for the public vaccination campaign, but rather can protect themselves from cholera if the vaccine is available in private market. The demand for vaccination further indicates that there is a potential scope for recovering a certain portion of the expenditure of immunization program by introducing direct user fees for future cholera vaccination in Bangladesh. A combination of funding from revenue of private market and pooled fund (e.g., taxes) could be considered as a sustainable way of financing oral cholera vaccine in Bangladesh to secure protection against cholera.

## Supporting information

S1 File Data collection tools. (DOCX)

## References

[1] Cai L, Modnak C, Wang J. An age-structured model for cholera control with vaccination. Applied Mathematics and Computation. Elsevier Inc.; 2017;299: 127–140. 10.1016/j.amc.2016.11.013

[2] Ali M, Nelson AR, Lopez AL, Sack DA. Updated global burden of cholera in endemic countries. PLoS neglected tropical diseases. 2015;9: 1–13. 10.1371/journal.pntd.0003832 26043000 PMC4455997

[3] IVI. Country investment case study on Cholera vaccination: Bangladesh. Seoul, South Korea; 2013.

[4] Alam M, Kasan NA, Sadique A, Bhuiyan NA, Ahmed KU, Nusrin S, et al Seasonal cholera caused by Vibrio cholerae serogroups O1 and O139 in the coastal aquatic environment of Bangladesh. Applied and Environmental Microbiology. 2006;72: 4096–4104. 10.1128/AEM.00066-06 16751520 PMC1489596

[5] Khan AI, Levin A, Chao DL, DeRoeck D, Dimitrov DT, Khan JAM, et al The impact and cost-effectiveness of controlling cholera through the use of oral cholera vaccines in urban Bangladesh: A disease modeling and economic analysis. PLoS neglected tropical diseases. 2018;12: e0006652 10.1371/journal.pntd.0006652 30300420 PMC6177119

[6] Sarker AR, Islam Z, Khan IA, Saha A, Chowdhury F, Khan AI, et al Cost of illness for cholera in a high risk urban area in Bangladesh: an analysis from household perspective. BMC Infectious Diseases. 2013;13: 518 10.1186/1471-2334-13-518 24188717 PMC4228304

[7] Chowdhury F, Rahman MA, Begum YA, Khan AI, Faruque ASG, Saha NC, et al Impact of Rapid Urbanization on the Rates of Infection by Vibrio cholerae O1 and Enterotoxigenic Escherichia coli in Dhaka, Bangladesh. PLOS Neglected Tropical Diseases. 2011;5. 10.1371/journal.pntd.0000999 PMC3071362 21483709

[8] WHO. Cholera vaccines: a new public health tool? Report of a WHO meeting 10–11 December 2002, Geneva. Geneva. Geneva Switzerland; 2004.

[9] Greenwood B. The contribution of vaccination to global health: Past, present and future. Philosophical Transactions of the Royal Society B: Biological Sciences. 2014;369 10.1098/rstb.2013.0220 PMC4024226 24821919

[10] Ozawa S, Mirelman A, Stack ML, Walker DG, Levine OS. Cost-effectiveness and economic benefits of vaccines in low- and middle-income countries: A systematic review. Vaccine. Elsevier Ltd; 2012;31: 96–108. 10.1016/j.vaccine.2012.10.103 23142307

[11] Stack ML, Ozawa S, Bishai DM, Mirelman A, Tam Y, Niessen L, et al Estimated economic benefits during the “decade of vaccines” include treatment savings, gains in labor productivity. Health Affairs. 2011;30: 1021–1028. 10.1377/hlthaff.2011.0382 21653952

[12] Levine OS, Bloom DE, Cherian T, De Quadros C, Sow S, Wecker J, et al The future of immunisation policy, implementation, and financing. The Lancet. 2011;378: 439–448. 10.1016/S0140-6736(11)60406-6 21664676

[13] Shen AK, Weiss JM, Andrus JK, Pecenka C, Atherly D, Taylor K, et al Country ownership and gavi transition: Comprehensive approaches to supporting new vaccine introduction. Health Affairs. 2016;35: 272–276. 10.1377/hlthaff.2015.1418 26858380

[14] Bärnighausen T, Bloom DE, Cafiero-Fonseca ET, O’Brien JC. Valuing vaccination. Proceedings of the National Academy of Sciences of the United States of America. 2014;111: 12313–9. 10.1073/pnas.1400475111 25136129 PMC4151736

[15] Kim S, Krishna H, Sagiraju R, Russell LB. Willingness-To-Pay for Vaccines in Low- and Middle-Income Countries: A Systematic Review. Annals of Vaccines and Immunization. 2014;1: 1–13.

[16] Carson RT. Contingent Valuation: A Practical Alternative When Prices Aren’t Available. Journal of Economic Perspectives. 2012;26: 27–42. http://www.aeaweb.org/jep/

[17] NOAA. Report of the NOAA panel on contingent valuation. National Oceanic and Atmospheric Administration Fed Reg- ist. 1993;58: 4607–14.

[18] Kobelt G. Health Economics: An Introduction to Economic Evaluation. 3rd ed. London, UK: Office of Health Economics; 2013.

[19] Yeung RYT, Smith RD. Can we use contingent valuation to assess the demand for childhood immunisation in developing countries? A systematic review of the literature. Applied Health Economics and Health Policy. 2005;4: 165–173. 10.2165/00148365-200504030-00005 16309334

[20] Johannesson M, Jönsson B. Economic evaluation in health care: is there a role for cost-benefit analysis? Health policy (Amsterdam, Netherlands). 1991 pp. 1–23. 10.1016/0168-8510(91)90114-d 10113574

[21] Drummond M, Sculpher MJ, Laxton KC, Stoddart GL, Torrance GW. Methods for the Economic Evaluation of Health Care Programmes. Third edit. Oxford University Press; 2005.

[22] Heinzen RR, Bridges JFP. Comparison of four contingent valuation methods to estimate the economic value of a pneumococcal vaccine in Bangladesh. International journal of technology assessment in health care. 2008;24: 481–7. 10.1017/S026646230808063X 18828944

[23] Randall A, Ives B, Eastman C. Bidding games for valuation of aesthetic environmental improvements. Journal of Environmental Economics and Management. 1974;1: 132–149. 10.1016/0095-0696(74)90010-2

[24] Kartman B, Stålhammar NO, Johannesson M. Valuation of health changes with the contingent valuation method: a test of scope and question order effects. Health economics. 1996;5: 531–541. 10.1002/(SICI)1099-1050(199611)5:6<531::AID-HEC235>3.0.CO;2-J 9003940

[25] Lichtenstein, Donald R, Bearden WO. Contextual Influences on Perceptions of Merchant-Supplied Reference Prices. Journal of Consumer Research. 1989; 55–66.

[26] Hoevenagel R. The contingent valuation method: scope and validity. Vrije Universiteit, University of Amsterdam 1994.

[27] O’Brien B, Goeree R, Gafni A, Torrance GW, Pauly M V., Erder H, et al Assessing the Value of a New Pharmaceutical: A Feasibility Study of Contingent Valuation in Managed Care. Medical Care. 1998;36: 370–384. 10.1097/00005650-199803000-00013 9520961

[28] O’Brien B, Viramontes JL. Willingness to Pay A Valid and Reliable Measure of Health State Preference? Medical Decision Making. 1994; 289–297. 10.1177/0272989X9401400311 7934716

[29] Anh DD, Canh DG, Lopez AL, Thiem VD, Long PT, Son NH, et al Safety and immunogenicity of a reformulated Vietnamese bivalent killed, whole-cell, oral cholera vaccine in adults. Vaccine. 2007;25: 1149–1155. 10.1016/j.vaccine.2006.09.049 17055622

[30] Mahalanabis D, Lopez AL, Sur D, Deen J, Manna B, Kanungo S, et al A randomized, placebo-controlled trial of the bivalent killed, whole-cell, oral cholera vaccine in adults and children in a cholera endemic area in Kolkata, India. PLoS ONE. 2008;3 10.1371/journal.pone.0002323 18523643 PMC2396289

[31] Saha A, Chowdhury MI, Khanam F, Bhuiyan MS, Chowdhury F, Khan AI, et al Safety and immunogenicity study of a killed bivalent (O1 and O139) whole-cell oral cholera vaccine Shanchol, in Bangladeshi adults and children as young as 1 year of age. Vaccine. Elsevier Ltd; 2011;29: 8285–8292. 10.1016/j.vaccine.2011.08.108 21907255

[32] WHO. Cholera. Fact sheet. 2016;

[33] Islam Z, Maskery B, Nyamete A, Horowitz MS, Yunus M, Whittington D. Private demand for cholera vaccines in rural Matlab, Bangladesh. Health Policy. 2007/09/08. 2008;85: 184–195. 10.1016/j.healthpol.2007.07.009 17822799

[34] Whittington D. Improving the Performance of contingent valuation studies in developing countries. Environmental and Resource Economics. 2002;22: 327–367.

[35] Suraratdecha C, Ainsworth M, Tangcharoensathien V, Whittington D. The private demand for an AIDS vaccine in Thailand. Health Policy. 2005;71: 271–287. 10.1016/j.healthpol.2004.05.005 15694496

[36] Canh DG, Whittington D, Thoa LTK, Utomo N, Hoa NT, Poulos C, et al Household demand for typhoid fever vaccines in Hue, Vietnam. Health Policy and Planning. 2006;21: 241–255. 10.1093/heapol/czl009 16581824

[37] Birhane MG, Miranda MEG, Dyer JL, Blanton JD, Recuenco S. Willingness to Pay for Dog Rabies Vaccine and Registration in Ilocos Norte, Philippines (2012). PLoS Neglected Tropical Diseases. 2016;10: 1–19. 10.1371/journal.pntd.0004486 26999021 PMC4801174

[38] Whittington D, Matsui-Santana O, Freiberger JJ, Van Houtven G, Pattanayak S. Private demand for a HIV/AIDS vaccine: evidence from Guadalajara, Mexico. Vaccine. 2002/06/12. 2002;20: 2585–2591. 10.1016/s0264-410x(02)00152-4 12057616

[39] Kairu-Wanyoike SW, Kaitibie S, Heffernan C, Taylor NM, Gitau GK, Kiara H, et al Willingness to pay for contagious bovine pleuropneumonia vaccination in Narok South District of Kenya. Preventive Veterinary Medicine. Elsevier B.V.; 2014;115: 130–142. 10.1016/j.prevetmed.2014.03.028 24774477 PMC4062942

[40] Weisberg S. Applied Linear Regression. 4th ed. Walter A, Samuel A, editors. Wiley; 2005.

[41] Beyer H. Tukey JW. Exploratory Data Analysis. Addison-Wesley Publishing Company Reading, Mass. Menlo Park, Cal., London, Amsterdam, Don Mills, Ontario, Sydney 1977, XVI, 688. S Biometrical Journal. 1981;23: 414.

[42] GoB. Bangladesh Economic Review 2015. Ministry of finance, Govornment of the people Republic of Bangladesh,Dhaka; 2015.

[43] Sauerborn R, Gbangou A, Dong H, Przyborski JM, Lanzer M. Willingness to pay for hypothetical malaria vaccines in rural Burkina Faso. Scandinavian journal of public health. 2005;33: 146–150. 10.1080/14034940510005743 15823976

[44] Botzen WJW, van den Bergh JCJM. Risk attitudes to low-probability climate change risks: WTP for flood insurance. Journal of Economic Behavior and Organization. Elsevier B.V.; 2012;82: 151–166. 10.1016/j.jebo.2012.01.005

[45] Harrison GW, Lau MI, Rutström EE. Estimating risk attitudes in Denmark: A field experiment. Scandinavian Journal of Economics. 2007; 10.1111/j.1467-9442.2007.00496.x

[46] Lucas MES, Jeuland M, Deen J, Lazaro N, MacMahon M, Nyamete A, et al Private demand for cholera vaccines in Beira, Mozambique. Vaccine. 2007/01/30. 2007;25: 2599–2609. 10.1016/j.vaccine.2006.12.027 17258844

[47] Dickinson KL, Hayden MH, Haenchen S, Monaghan AJ, Walker KR, Ernst KC. Willingness to pay for mosquito control in key west, Florida and Tucson, Arizona. American Journal of Tropical Medicine and Hygiene. 2016;94: 775–779. 10.4269/ajtmh.15-0666 26903603 PMC4824217

[48] Harapan H, Anwar S, Bustamam A, Radiansyah A, Angraini P, Fasli R, et al Willingness to pay for a dengue vaccine and its associated determinants in Indonesia: A community-based, cross-sectional survey in Aceh. Acta Tropica. Elsevier B.V.; 2017;166: 249–256. 10.1016/j.actatropica.2016.11.035 27908746

[49] Palanca-Tan R. The demand for a dengue vaccine: A contingent valuation survey in Metro Manila. Vaccine. 2008;26: 914–923. 10.1016/j.vaccine.2007.12.011 18206277

[50] Harapan H, Mudatsir M, Yufika A, Nawawi Y, Wahyuniati N, Anwar S, et al Community acceptance and willingness-to-pay for a hypothetical Zika vaccine: A cross-sectional study in Indonesia. Vaccine. 2019; 10.1016/j.vaccine.2019.01.062 30739794

[51] Pachico D, Wolf MM. Consumer Acceptance of Genetically Modified Foods. Evenson RE, Santaniello V, editors. Consumer acceptance of genetically modified foods. Oxon, UK: Library of Congress Cataloging-in-Publication Data; 2009 10.1079/9780851997476.0155

[52] Kim D, Canh DG, Poulos C, Thoa LTK, Cook J, Hoa NT, et al Private demand for cholera vaccines in Hue, Vietnam. Value in Health. 2008;11: 119–128. 10.1111/j.1524-4733.2007.00220.x 18237366

[53] MOHFW. Bangladesh National Health Accounts 1997–2012. Dhaka, Bangladesh; 2015. 10.13140/RG.2.1.3951.6247

[54] Sarker AR, Islam Z, Khan IA, Saha A, Chowdhury F, Khan AI, et al Estimating the cost of cholera-vaccine delivery from the societal point of view: A case of introduction of cholera vaccine in Bangladesh. Vaccine. Elsevier Ltd; 2015;33: 4916–4921. 10.1016/j.vaccine.2015.07.042 26232545

[55] Incepta. Steering committee meeting of oral Cholera vaccine (OCV) in Bangladesh. 2017.

[56] Ali M, Emch M, Von Seidlein L, Yunus M, Sack DA, Rao M, et al Herd immunity conferred by killed oral cholera vaccines in Bangladesh: A reanalysis. Lancet. 2005; 10.1016/S0140-6736(05)66550-6 15993232

[57] Klose T. The contingent valuation method in health care. Health Policy. 1999;47: 97–123. 10.1016/s0168-8510(99)00010-x 10538292

[58] Jeuland M, Lucas M, Clemens J, Whittington D. Estimating the private benefits of vaccination against cholera in Beira, Mozambique: A travel cost approach. Journal of Development Economics. Elsevier B.V.; 2010;91: 310–322. 10.1016/j.jdeveco.2009.06.007

