# Supplementary material for: Willingness to pay for oral cholera vaccines in urban Bangladesh
Source: PLoS One. 2020 Apr 30;15(4):e0232600. doi: 10.1371/journal.pone.0232600 (PMC7192494; doi:10.1371/journal.pone.0232600)
Supplement: S1 File — (DOCX) [file pone.0232600.s001.docx]

## Data Collection tool:

## Study on willingness to pay for oral cholera vaccine

**Section 1: Background Information**

PID Number: |____|____|____|____|____|____|____|____|____|____|____|

Name of the respondent: __________________________________________________________________________

Address: _____________________________________________________________

_____________________________________________________________

Age the respondent: Year |____|____| Month |____|____|

**Relation:** Mother (1), Father (2), Brother (3), Sister (4), Grandparents (5), Relative (6), Neighbour/ Friend (7), Other (8)

Relationship with the Household’s member (insert code): |_______|

Contact no (Cell/Telephone) |____|____|____|____|____|-|____|____|____|____|____|____|

The above cell phone is belonging to you? Yes (1) No (2)

Interviewer ID: |______|______|______|______|______|______|

Interview start time: |__|__|:|__|__| (HH:MM) AM PM

Interview end time: |__|__|:|__|__| (HH:MM) AM PM

Interviewer, how long did you have to wait, after arriving at the respondent's home, to conduct this interview? (Please fill in below in minutes, if you didn't have to wait, please record "zero") ___________ Minute

If the survey is not completed, please indicate the reason why?

(1) Respondent seriously ill, cannot reschedule

(2) Respondent refused to be interviewed

(3) Respondent refused to sign consent

(4) Respondent decided to stop before finishing interview

(5) Respondent absent

(6) No children under 15 yrs. in the family

(99) Others, specify

| 201..Member ID | 202. Name | 203. Relationship with the household head | 204. Sex Male (1), Female (2) | 205. Age (actual) | | 206.Marital status | | 207. Education (complete year ) | 208. Occupation | 209. How many days work in last 1 month | 210. Monthly income (e.g. consider last month) |
| --- | --- | --- | --- | --- | --- | --- | --- | --- | --- | --- | --- |
|  |  |  |  |  |  |  |  |  | Code of occupation 1. Farmer  2. Rickshaw/ van driver  3. Bus/ truck/ CNG driver  4. Garments/factory worker  5. Fisherman  6. Public Job  7. Private Job  8. Business  9. Tea stall  10. Daily worker  11. Farm house  12. Fishmonger  13. Restaurant  14. Small scale  15. Homemaker 16. Domestic worker 17. Immigrant 18. Student 19. Unemployed 20. Disable person 21. Beggar 22. Retired  88. Other. 99. Not applicable |  |  |
|  |  |  |  |  |  | 1 | Married |  |  |  |  |
|  |  |  |  |  |  | 2 | Unmarried |  |  |  |  |
|  |  |  |  |  |  | 3 | Widow |  |  |  |  |
|  |  |  |  |  |  | 4 | Divorce |  |  |  |  |
|  |  |  |  |  |  | 5 | Separation |  |  |  |  |
|  |  |  |  |  |  |  | |  |  |  |  |
|  |  |  |  |  |  |  |  |  |  |  |  |
|  |  |  |  |  |  |  |  |  |  |  |  |
|  |  |  |  |  |  |  |  |  |  |  |  |
|  |  |  |  |  |  |  |  |  |  |  |  |
|  |  |  |  |  |  |  |  |  |  |  |  |
|  |  |  |  |  |  |  |  |  |  |  |  |
|  |  |  |  | Year | Month |  | |  |  |  |  |
|  |  |  |  |  |  |  |  |  |  |  |  |
|  |  |  |  |  |  |  |  |  |  |  |  |
| 1 |  |  |  |  |  |  | |  |  |  |  |
| 2 |  |  |  |  |  |  | |  |  |  |  |
| 3 |  |  |  |  |  |  | |  |  |  |  |
| 4 |  |  |  |  |  |  | |  |  |  |  |
| 5 |  |  |  |  |  |  | |  |  |  |  |
| 6 |  |  |  |  |  |  | |  |  |  |  |
| 7 |  |  |  |  |  |  | |  |  |  |  |
| 8 |  |  |  |  |  |  | |  |  |  |  |
| 9 |  |  |  |  |  |  | |  |  |  |  |

| 211. Did you/ your family member spend any money due to receiving healthcare? | | | | Yes . . . . (1)  No . . . . (2) 213 | | | | |
| --- | --- | --- | --- | --- | --- | --- | --- | --- |
| 212. If yes, please specify | | | | Item | | Amount ( BDT) | | |
|  |  |  |  | Medicine | |  | | |
|  |  |  |  | Physician fee | |  | | |
|  |  |  |  | Diagnosis | |  | | |
|  |  |  |  | Bed Fee | |  | | |
|  |  |  |  | Food | |  | | |
|  |  |  |  | Transport/ communication | |  | | |
|  |  |  |  | Other (specify) | |  | | |
|  |  |  |  | Total | |  | | |
| 213. Have you heard anything about this study from your friend, neighbors, or family members? | | | | Yes . . .(1)  No . . . .(2)  If yes specify _________________________________________ | | | | |
| **Section -3. Perceptions and Attitude Towards Cholera** | | | | | | | | |
| The next questions I would like to ask you are about the disease cholera. | | | | | | | | |
| 301. Have you ever heard of the disease cholera? | | | | Yes . . . . . . . . . . . .(1)  No . . . . . . . . . . . .(2)  Don't know/not sure. . (99) | | | | |
| 302. What are the symptoms of cholera?  (Spontaneous response, more than one response permitted) | | | | *Please read the following description to all respondents:*  Cholera is a disease often characterized by severe diarrhea, frequent episodes of watery diarrhea, vomiting, and weakness | | | | |
| Don't know/not sure | | | | 99 | | | | |
| 303. How does someone become infected by cholera? (Spontaneous response, more than one response permitted: check all that apply) | | | | | | | | |
| *Answer* | | | *Please mark (circle) is applicable* | | | | | |
| drinking unboiled water | | | 1 | | | | | |
| eating food from street vendors | | | 2 | | | | | |
| eating unclean, uncooked vegetables | | | 3 | | | | | |
| eating unripe fruit | | | 4 | | | | | |
| bad weather | | | 5 | | | | | |
| using unhygienic latrines | | | 6 | | | | | |
| not washing hands before/after eating | | | 7 | | | | | |
| flies touching food | | | 8 | | | | | |
| Outdated food | | | 9 | | | | | |
| Others (specify) | | | 88 | | | | | |
| don't know/not sure | | | 99 | | | | | |
| 304. How common do you think cholera is in your neighborhood? (read all responses before taking answer; one response permitted) | | | Not very common . . . . . . . (1)  Common . . . . . . . . . (2)  Very Common . . . . . . . . . . (3)  Don't know/not sure . . . (99) 306 | | | | | |
| 305. How serious is cholera for the following groups? (For each group, read all responses and mark one response | | | Age range | | Code | | Very serious (1)  Serious (2)  Not so serious (3)  Don't know/not sure (99) | |
|  |  |  | Members aged under 5 years and | |  | |  |  |
|  |  |  | Members aged 6 to 10 years | |  | |  |  |
|  |  |  | Members aged 11to14 years | |  | |  |  |
|  |  |  | Members aged 15-19 years and above | |  | |  |  |
|  |  |  | Members aged 20 to 64 years and above | |  | |  |  |
| 306. Has anybody in your household (including yourself) ever had cholera? | | | Yes . . . . . . . . . . . (1)  No . . . . . . . . . . . .(2)  Don't know/not sure. (99) | | | | | |
| 307. Has anybody in your household ever died due to cholera? | | | Yes . . . . . . . . . . . (1)  No . . . . . . . . . . . .(2) 309  Don't know/not sure. (99) | | | | | |
| 308. For each person in your household who died of cholera, please tell me how old they were when they died. (Spontaneous response; record total number of individuals who died of cholera in each group) | | | | | | | | |
| List of household member | | | | Number | | | | |
| Infant less than one | | | |  | | | | |
| Members aged under 1 to 5 years and | | | |  | | | | |
| Members aged 6 to 10 years | | | |  | | | | |
| Members aged 11to14 years | | | |  | | | | |
| Members aged 15-19 years and above | | | |  | | | | |
| Members aged 20 to 64 years and above | | | |  | | | | |
| 309. Have you known personally anyone (other than a household member) who has been sick due to cholera? | | | | Yes . . . . . . . . . . . (1)  No . . . . . . . . . . . .(2)  Don't know/not sure. (99) | | | | |
| 310. Have you known personally anyone (other than a household member) who has died due to cholera? | | | | Yes . . . . . . . . . . . (1)  No . . . . . . . . . . . .(2)  Don't know/not sure. (99) | | | | |
| **Section 4. Vaccines and Cholera** | | | | | | | | |
| Next I'd like to talk about the spread and prevention of cholera. Cholera is spread primarily through eating food and drinking water contaminated by the feces of patients. You can help protect yourself from cholera by always consuming only safe, clean food and water and washing your hands thoroughly after defecation and before taking food.  Cholera is caused by a type germ. When someone becomes ill with cholera, he/she can develop severe diarrhea that can cause him or her to lose large amounts of fluids and salts. When the body loses too many fluids and salts, it can no longer work properly. The patient's kidneys can stop working, and the patient could die. The patient with cholera should drink plenty of oral saline and when severe, take intravenous saline/ cholera saline. If the patient takes Antibiotics right away, the diarrhea should not last as long.  The diarrhea caused by cholera will stop in a few days. Giving fluids works well to prevent and treat the worst problems caused by cholera, and giving fluids also makes the patient feel better. However, without treatment a person with cholera can become severely sick or die. | | | | | | | | |
| 401. Do you have any questions or anything you are not clear about | | | | Yes . . . (1)  No . . . (2) 402 | | | | |
| If yes, record the respondent's questions:  *____________________________________________________________*  *_____________________________________________________________________*  *[Enumerator: If you know the answer to the respondent's questions, please answer them truthfully and briefly. If you are not sure you know the answer, please tell the respondent that you are not sure.]* | | | | | | | | |
| ***I would like to ask you the following questions about vaccines.*** | | | | | | | | |
| 402. Have you ever heard about vaccines? | | | | Yes . . . (1)  No . . . (2) | | | | |
| 403. In your opinion, what is the purpose of a vaccine? (Spontaneous response, multiple response permitted) | | | | | | | | |
| Prevent disease for children . . . . . . . . . . .(1)  Prevent disease for pregnant women . . . . ...(2)  Prevent disease for all people . . . . . . . . . .(3)  Cure disease . . . . . . . . .. . . . . . .. . . ..(4)  Others (specify) . . . . . . . . . . . . . . . .(88)  Don't know/not sure . . . . . . . . . . . . . . . . (99) | | | |  | | | | |
| Read the following statement to all responded  ***Vaccine is for "prevention", not for treatment. You have to take a vaccine before you get sick.*** | | | | | | | | |
| 404. Have you been vaccinated before? | | | | Yes . . . . . . . . . . . . (1)  No . . . . . . . . . . . . (2)  Dont know/ Not sure . (99) | | | | |
| 405. Has anyone in this household including you had either the any cholera vaccine? | | | | Yes . . . . . . . . . . . . . .(1)  No . . . . . . . . . . . . . . (2) 409  Dont know/ Not sure . . . (99) | | | | |
| 406. If yes, from where you received/ bought? | | | | Pharmacy . . . . . . . . . . .(1)  Private hospital . . . .(2)  Public hospital . . . .(3)  Vaccination centre . . . . . . .(4)  Other (specify) ___(88) | | | | |
| 407. Were you satisfied with that vaccine? | | | | Yes . . . . . . . . . . . . . . . .(1) 409  No . . . . . . . . . . . . . . . . (2)  Dont know/ Not sure . . . . . (99) | | | | |
| 408. If no, why not? (Spontaneous response, record only the most important) [Enumerator: If the respondent gave more than one reason, please ask which is the most important reason] | | | | Did not prevent Cholera____ (1)  Was not satisfied with the characteristics of vaccine (i.e. smell or color or taste)____(2)  not satisfied with the method of administering the vaccine____(3)  Minor side effects (i.e. diarrhea, rash, leaves scars on skin, fever, headache, loss of appetite, vomiting)____(4)  Caused other major health problems ______ (5)  Because the vaccine was locally produced _____(6)  Other specify _______________ (88)  Dont know/ Not sure _______________99) | | | | |
| 409. Do you think that the vaccine will work against cholera disease? | | | | Yes . . . . . . . . . . . . (1)  No . . . . . . . . . . . . (2)  Dont know/ Not sure . (99) | | | | |
| 410. In your household, who would be primarily involved in making the decision whether or not to purchase cholera vaccines for your household members? (Spontaneous response, multiple responses permitted) | | | | Myself (respondent . . . . . .(1)  Spouse of respondent . . . . . . (2)  Parents of respondent . . . . . . . . .(3)  Parents in-law(s) of respondent . . . . . .(4)  Son/ Daughter of respondent . . . . . . . . . .(5)  Other (specify) . . . . . . . (88)  Dont Know/Not sure . . . . . . . . .(99) | | | | |
| **Section 5. Cholera Vaccine (CV) scenario** | | | | | | | | |
| Please explained the following topics in detailed | | | | | | | | |
| Doctors and scientists have developed a new vaccine that can prevent people from getting cholera. We'd like to know what you would do if the new cholera vaccine was available for sale at a convenient location like a vaccination camp or vaccination clinic or in any private clinic or pharmacy.   - This new vaccine could be given to individuals to prevent them from having cholera in the future - This vaccine is completely safe and has no side effect and orally administrated like polio vaccine - It could not be used to treat someone who currently has cholera. - This vaccine cannot be used for children under 1 year and pregnant women. - the vaccine would be required taken about 2 weeks apart - The vaccine will upto 60% effective for 2 years duration | | | | | | | | |
| **Vaccine Effectiveness** | | | | | | | | |
| Now I want to explain exactly what I mean when I say the vaccine would be [60%] effective. Suppose that each of these little blue or red figures (*Enumerator: show the picture*) represents a person. (Enumerator: point out the circle). The 100 figures inside this circle represent 100 persons who have taken the vaccine, while the figures outside the circle represents those who have not taken the vaccine. The cholera vaccine is not 100% effective; that is the vaccine is only (60%) effective. Therefore, of the 100 people taking the vaccine in the circle, there will be (60%) of the people who have taken the vaccine that are protected (i.e., the vaccine works for them) for a period of 2 years. The blue figures inside this circle represent these people.  The rest of the people (the red ones inside the circle) who have been vaccinated (40) will not be protected against cholera even though they have taken the vaccine, because the vaccines did not work for them. They will still be at risk of getting cholera just like they were before they got the vaccine or just like the people outside the circle who haven't received vaccines. However, even if they get cholera, their symptoms may not be quite as severe compared to someone who has not received the vaccine.  The people who receive cholera vaccine will not be able to know if the vaccine works for them. Of course, we don't know who would actually get cholera. A red person outside the circle who has not taken a vaccine still has a relatively small risk of being infected. | | | | | | | | |
| **Assess understanding about the vaccine effectiveness**  Now I am going to ask you some questions to make sure that the information I told you is clear | | | | | | | | |
| **First round** | | | | | | | | |
| 501. Please point to all the people who have taken the vaccine [Interviewer: put a mark into a relevant place] | | | | Respondent did give the correct answer____(1)  Respondent did not give the correct answer ___(2)  Respondent did not know/not sure____(3) | | | | |
| 502. Please point to all the people who have taken the vaccine and it work for them. [Interviewer: put a mark into a relevant place] | | | | Respondent did give the correct answer____(1)  Respondent did not give the correct answer ___(2)  Respondent did not know/not sure____(3) | | | | |
| 503. How many years would the cholera vaccine work for them? | | | | Year_____ (1)  If respondent gave incorrect answer, please correct it (2)  Respondent did not know/not sure____(3) | | | | |
| 504. How many people have taken the vaccine but can still get cholera? [*Interviewer: put a mark into a relevant place*] | | | | Respondent did give the correct answer____(1)  Respondent did not give the correct answer ___(2)  Respondent did not know/not sure____(3) | | | | |
| 505.If an unvaccinated person gets infected by cholera, can the vaccine be used to cure them? | | | | Respondent did give the correct answer____(1)  Respondent did not give the correct answer ___(2)  Respondent did not know/not sure____(3)  *If respondent gave incorrect answer, please correct it.* | | | | |
| 506. Interviewer: did the respondent give the correct answer to all three effectiveness questions (501,502 and 504) | | | | Yes . . . . . . (1) 513  No. . . . . . .(2)  *Enumerator: If No to* ***CV Scenario*** *tell the respondent:* | | | | |
| *"I feel that I need to explain about the effectiveness of the vaccine a little bit more." (explain the effectiveness of the vaccine again) "Now I would like to go over the questions again, to make sure that the information I told you is clear."* | | | | | | | | |
| **Second round** | | | | | | | | |
| 507. Please point to all the people who have taken the vaccine [Interviewer: put a mark into a relevant place] | | | | Respondent did give the correct answer____(1)  Respondent did not give the correct answer ___(2)  Respondent did not know/not sure____(3) | | | | |
| 508. Please point to all the people who have taken the vaccine and it work for them. [Interviewer: put a mark into a relevant place] | | | | Respondent did give the correct answer____(1)  Respondent did not give the correct answer ___(2)  Respondent did not know/not sure____(3) | | | | |
| 509. How many years would the cholera vaccine work for them? | | | | Year_____ (1)  If respondent gave incorrect answer, please correct it (2)  Respondent did not know/not sure____(3) | | | | |
| 510. How many people have taken the vaccine but can still get cholera? [*Interviewer: put a mark into a relevant place*] | | | | Respondent did give the correct answer____(1)  Respondent did not give the correct answer ___(2)  Respondent did not know/not sure____(3) | | | | |
| 511. If an unvaccinated person gets infected by cholera, can the vaccine be used to cure them? | | | | Respondent did give the correct answer____(1)  Respondent did not give the correct answer ___(2)  Respondent did not know/not sure____(3)  *If respondent gave incorrect answer, please correct it.* | | | | |
| 512. Interviewer: did the respondent give the correct answer to all three effectiveness questions (507, 508 and 510) | | | | Yes . . . . . . (1) 513  No. . . . . . .(2)  *Enumerator: If No to* ***CV Scenario*** *tell the respondent:* | | | | |
| Note: Whether the respondents gave the correct answer or not, please skip to the next question. | | | | | | | | |
| 513. Please indicate what you believe to be the most important benefit of the vaccine. | | | | | | | | |
| Prevent pain and suffering of cholera . . . . . . . . . . . . . (1)  Avoid teatment cost of cholera . . . . . . (2)  Prevent risk of death from cholera . . . . . .(3)  Avoid income loss due to cholera . . . . . .(4)  Don’t know/No answer . . . . (5)  Othjer ( please specify) . . . . . . . . . . . . . . . . . . . . (99) | | | |  | | | | |
| 514. With this information, will you be willing to accept Cholera vaccine if it is offered in any immunization facility? | | | | Yes . . . . .(1)  No . . . . ..(2) | | | | |
| 515. Please rate your level of acceptance of Cholera vaccine? | | | | Very unwilling . . . . (1)  Unwilling . . . . . . . . (2)  Not sure (3)...  Willing . . . . . . . . (4)  Very willing . . . ... .(5) | | | | |
| **Section 6. Willingness to pay for Cholera Vaccine** | | | | | | | | |
| Suppose, the government will not provide the vaccine at free. If someone wish to vaccinated, he/she must purchase the vaccine with specified price. The purchasing price of the vaccine will be uniform to all. In this situations, Now I'd like to know whether you would buy the vaccine if it was available at a specified price. Some people say they cannot afford the price of the vaccine or that they are actually not at risk of getting this disease. Other people say that would buy the vaccine because the protection is really worth it to them. Again some other people replied, “this vaccine has a great importance and I would really like as much protection from this disease as possible."  In other studies about vaccines, we have found that people sometimes say they want to buy the vaccine. They think: "I would really like as much protection from this disease as possible." However, they may forget about other things they need to spend their money on in real life. Please try to think carefully about what you would actually do if you had to spend your own money. There are no rights or wrong answers. We really want to know what you would do.  For your information, a new cholera vaccine named “ Shancol” is available in india at a price ranges between 127-150 Bangladeshi taka, i.e., a total BDT 254-300 will be required for two dose. However, a study conducted in urban Bangladesh and found that, the average total household cost of treatment for an episode of cholera was up to BDT 2278 which is quite high. | | | | | | | | |
| **Willingness to Pay (WTP) (FOR SELF)** | | | | | | | | |
| 601. If the Cholera vaccine is not publicly funded, will you be willing to pay for it for two dose? | | | | Yes . . . (1) 603  No . . . (2) 602 and 605 থেকে continue | | | | |
| 602. If no, Why?  (Spontaneous response, multiple responses permitted) | | | | Not enough money. . . . . . . . . . . . .. . . . . .(1)  Too expensive. . . . . . . . . . . . . . . . . . . . . . (2)  I am too old a not required. . . . . . . . (3)  Not for myself but only for children. . . . . . . (4)  Yes, only if the doctor recommends. . . . . . . . . . .(5)  Yes, only if many people around me get cholera illness .(6)  Respondent did not know/not sure . . . (99) | | | | |
| 603. How much will you be willing to pay for the two dose cholera vaccine? | | | | \|______________________\| BDT | | | | |
| 604. If due to inflation or other uncertainties, the cost for the vaccine is higher than what you have just stated, what is the maximum amount you are very certain to pay for yourself? Bearing in mind that your entire household (both adult and children) may have to receive the vaccine about the same period? | | | | \|______________________\| BDT | | | | |
| **WTP (WTP for other household member)** | | | | | | | | |
| 605. If the Cholera vaccine is not publicly funded, will you be willing to pay for it for your household members? | | | | Yes . . . . .(1) 607 continue  No . . . . . (2) 606 & Section **7** continue | | | | |
| 606. If no, Why?  (Spontaneous response, multiple responses permitted) | | | | Not enough money . . . . . . . . . . . . . . . . . . . (1)  Too expensive . . . . . . . . . . . . . . . . . . . .(2)  Not for myself but only for children . . . . . . . . .(3)  Yes, only if the doctor recommends . . . . . . . . . (4)  Yes, only if many people around me get cholera illness. . (5)  Other (specify) _______________ . . . . . . . . .(88)  Respondent did not know/not sure . . . . . (99) | | | | |
| 607. Please fill up the following table (ONLY FOR HOUSEHOLD MEMBERS NOT INCLUDING YOURSELF) | | | | | | | | |
| Relationship | Yes (1), No (2) | Age | | Number of Dose | | | | Maximum WTP |
| Spouse |  |  | |  | | | |  |
| Mother |  |  | |  | | | |  |
| Father |  |  | |  | | | |  |
| Child 1 |  |  | |  | | | |  |
| Child 2 |  |  | |  | | | |  |
| Child 3 |  |  | |  | | | |  |
| Child 4 |  |  | |  | | | |  |
| Child 5 |  |  | |  | | | |  |
| Child 6 |  |  | |  | | | |  |
| Other adult 1 |  |  | |  | | | |  |
| Other adult 2 |  |  | |  | | | |  |
| Other adult 3 |  |  | |  | | | |  |
| Other Child 1 |  |  | |  | | | |  |
| Other Child 2 |  |  | |  | | | |  |
| 608. How difficult did you find it to make your decision? | | | | Very difficult . . . .(1)  Difficult . . . . . . (2)  Easy . . . . . . . .(3)  Very easy . . . . . (4) | | | | |
| **Section 7. End of questionnaire** | | | | | | | | |
| This is the end of the interview. Thank you very much for your participation. We’d like to state that it is necessary for you to protect yourself from contracting cholera. The objective of this survey is to learn about your willingness to pay for cholera vaccines either for yourself or your household members. We need to ask different households their willingness to purchase at different prices. Thus, don’t worry if you hear that other people in your community have been asked related to this survey | | | | | | | | |
| 701. How reliable do you think is the information you got from the respondent?? | | | | Very reliable . . . . . .(1)  Reliable . . . . . . . . . (2)  Fairly reliable . . . .(3)  Not reliable . . . . . . . (4)  Very unreliable. . (5) | | | | |
| 702. Do you think the respondent understood about the vaccine efficacy scenario and the importance of the vaccination? | | | | Did not understand . . . . .(1)  Fairly understood . . . . . . .(2)  Understood . . . . . . . . . . . .(3)  Don’t know/not sure . . .(99) | | | | |
| 703.  *Enumerator: Please note the type of flooring material* | | | | Mud . . . . . . . . . . . . . . (1)  Cement . . . . . . . . . . . . .(2)  Mosaic[floor tiles] .(3)  Brick . . . . . . . . . . . . . . .(4)  Others (specify) . . . (88) | | | | |
| 704.  *Enumerator: Please note the type of material used in the Wall* | | | | Thatch/ bamboos . . .. (1)  Mud . . . . . . . . . . . . . (2)  Corrugated tin . . ... (3)  Plastic/polythene.. . (4)  Bricks . . . . . . . . .. . . (5)  Wood . . . . . . . . . . . . (6)  Other (specify) . . . .(77) | | | | |
| 705.  *Enumerator: Please note the type of material used in the Roof* | | | | Thatch/bamboo/wood etc . . .(1)  Plastic/polythene . . . . . . . . (2)  Corrugated tin . . . . . . . . . . . . .(3)  Concrete . . . . . . . . . . . . .(4)  Others, Specify . . . .(77) | | | | |
| 706. What type of house does the respondent live in? | | | | Own homestead . . . . . . . . . . . (1)  Rented house in slum . . . . . . . . . . (2)  Government quarters . . . . . . . . . . . (3)  Single-family home in good condition . . . . .(4)  Flat/home shared by multiple families . . . . . (5)  Single-family home in poor condition . . . . (6)  Others, (specify) . . . . . . . .(77) | | | | |
| 707. Other suggestions/ comments...... | | | | | | | | |
